# MiR-181a-5p promotes anoikis by suppressing autophagy during detachment induction in the mammary epithelial cell line MCF10A

**DOI:** 10.1007/s13238-016-0255-8

**Published:** 2016-03-14

**Authors:** Jia-li Wei, Yuan-cheng Li, Zhong-liang Ma, You-xin Jin

**Affiliations:** School of Life Sciences, Shanghai University, Shanghai, 200444 China; Suzhou Institute of Nano-Tech and Nano-Bionics (SINANO), Chinese Academy of Sciences, Suzhou, 215123 China

**Dear Editor,**

MicroRNAs (miRNAs) are small non-coding RNAs of ~22 nucleotides (nt) in length that bind to the 3′UTRs of their target mRNAs, leading to translational inhibition or mRNA degradation (Bartel, 2009). miRNAs regulate the expression of hundreds of genes involved in various biological processes, such as apoptosis, migration, metastasis, and autophagy (Flynt and Lai, 2008; Frankel and Lund, 2012; Ventura and Jacks, 2009). Previous studies have indicated that miRNAs may act as oncogenes or antioncogenes in tumorigenesis of various cancers (Hammond, 2015).

When considering the rate of failure in cancer treatment, metastasis has been pinpointed as a major cause (Nguyen and Massague, 2007). Whereas, anoikis, a type of programmed cell death resulted from the loss of cell-matrix interactions, can suppress tumor metastases. In contrast, resistance to anoikis will enhance metastases by enabling ECM-detached cancer cells to survive (Frisch and Francis, 1994).

Recently, it has been reported that autophagy promoted cell survival during detachment-induced anoikis (Fung et al., 2008). In this study, we show that miRNA miR-181a-5p modulates anoikis in human mammary epithelial cell line (MCF10A); specifically, our data demonstrate that autophagy was significantly impaired in MCF10A cells stably-expressing miR-181a-5p, and that the effects of miR-181a-5p on anoikis were reversed by overexpression of autophagy-related gene, *ATG5*. In summary, we report a novel regulation of anoikis that involves the repression of autophagy by miR-181a-5p.

The expression of miR-181a-5p was upregulated during anoikis in mammary epithelial cell line MCF10A and breast cancer cell line MCF7. We examined the expression of dozens of miRNAs during anoikis in MCF10A cell line using quantitative real-time PCR and found that the expression levels of four miRNAs (miR-181a-5p, miR-143, miR-30b, and miR-338-5p) were upregulated. Notably, the expression level of miR-181a-5p showed the most significant increase. Furthermore, the expression of miR-181a-5p was detected in both MCF10A cell line and the breast cancer cell line MCF7 at 24 h and 48 h after detachment induction. In MCF10A cells, the expression of miR-181a-5p declined obviously at 24 h after detachment induction but increased at 48 h (Fig. 1A). However, in MCF7 cell line, the expression of miR-181a-5p increased at both 24 h and 48 h after detachment induction (Fig. 1B). These data indicate that the expression of miR-181a-5p can be modulated in the detachment environment.

**Figure 1:**
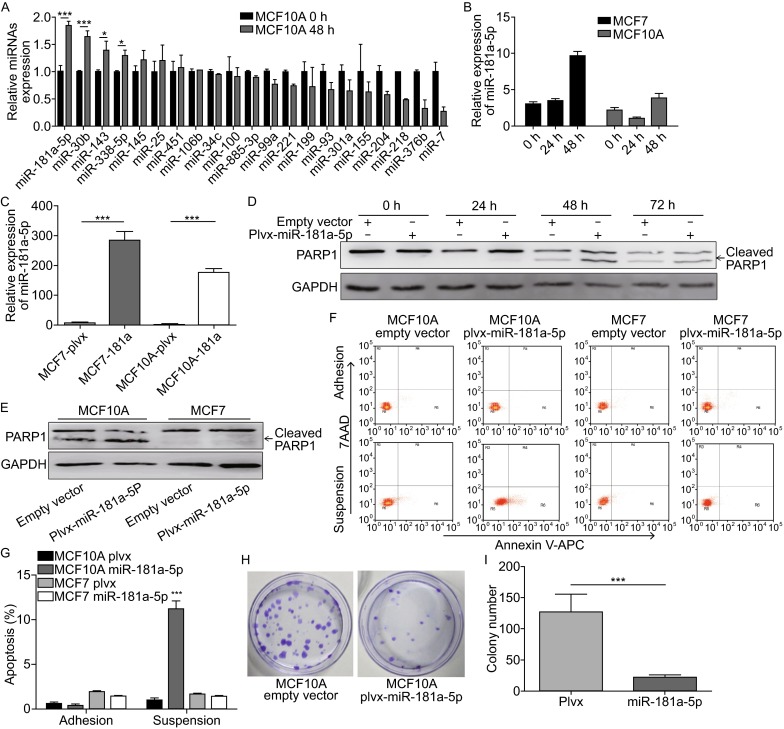
**Overexpression of miR-181a-5p accelerates the apoptosis of suspension MCF10A cells**. (A) The expression level of different miRNAs was measured in attached and detached (48 h) condition by QPCR assay. (B) miR-181a-5p expression level was detected under control (0 h) and anoikis (24 h and 48 h) conditions both in MCF10A and MCF7 cell lines. (C) QPCR analysis of miR-181a-5p expression levels in MCF10A and MCF7 stable cell lines. (D) Western blot analysis was performed to detect PARP1 protein levels at 0 h, 24 h, 48 h, and 72 h of anoikis induction. (E) PARP1 protein levels in MCF10A and MCF7 cells were detected after 48 h of suspension culture. (F) The apoptotic rate was detected with the Annexin V-APC kit following 48 h of suspension culture. For each cell line, 10,000 cells were counted by flow cytometry. (G) The percentage of apoptosis is plotted in a bar graph. A significant difference between MCF10A-plvx cells and MCF10A miR-181a-5p cells is indicated. The data correspond to the mean ± SD of three independent experiments. (H) Colony formation assay with cells cultured for 48 h in suspension conditions. Cells were stained with crystal violet. (I) The colony number was plotted in bar graph. *P < 0.05; ***P < 0.001

Overexpression of miR-181a-5p attenuated anoikis resistance. To examine the role of miR-181a-5p during cell detachment, we generated MCF10A and MCF7 cell lines stably expressing miR-181a-5p using a lentivirus vector containing a precursor miR-181a-5p expression cassette. As shown in Fig. 1C, the expression of miR-181a-5p increased by 175 folds in MCF10A cells stably-expressing miR-181a-5p, compared with the cells transfected with the empty vector (herein termed as control cells). PARP1 is one of the major cleavage targets of caspase-3 and it has been shown that cleavage of PARP1 promotes apoptosis (Soldani and Scovassi, 2002). Therefore, we examined the expression of cleaved PARP1 in MCF10A cells overexpressing miR-181a-5p and found that 48 h after detachment induction, expression levels of cleaved PARP1 were increased significantly compared with that in control cells; however, we did not observe any significant change in MCF7 cells stably-expressing miR-181a-5p (Fig. 1D and 1E). Next, we performed FACS analysis to examine the rate of apoptosis in MCF10A cells overexpressing miR-181a-5p 48 h after detachment induction (Fig. 1F and 1G). This data revealed that the rate of apoptosis in miR-181a-5p overexpressing MCF10A cells increased significantly compared with that in MCF10A control cells (Fig. 1F and 1G). Nonetheless, there was no significant difference in the rate of apoptosis between the miR-181a-5p overexpressing cells and the control cells in attachment culture. In addition, we performed colony formation assays and found that after 48 h in detachment culture followed by two weeks of attachment culture, the MCF10A cells overexpressing miR-181a-5p formed fewer colonies than the control cells (Fig. 1H and 1I). These results imply that miR-181a-5p could play an important role in suppression of anoikis resistance in MCF10A cells.

During anoikis, miR-181a-5p repressed autophagy. It has been reported that miR-181a-5p attenuated starvation-induced autophagy in MCF7 cells (Tekirdag et al., 2013) and previous studies suggest that autophagy is induced in detachment conditions to promote cell survival (Fung et al., 2008). Thus, we speculate that miR-181a-5p may inhibit anoikis resistance by blocking autophagy. To prove this hypothesis, we first examined the effects of miR-181a-5p on autophagy. As shown in Fig. 2A, the expression of LC3II increased after substratum detachment induction in MCF10A control cells, whereas the turnover of LC3II was obviously blocked in MCF10A cells stably-expressing miR-181a-5p. We did not observe any significant changes in the expression level of LC3II in MCF7 cells in both attachment and detachment conditions. In addition, miR-181a-5p has no effects on the expression of LC3II in MCF7 cells overexpressing miR-181a-5p (Fig. 2B). To further confirm the decrease of autophagosomes in MCF10A cells stably-expressing miR-181a-5p, we employed an immunofluorescence approach to assess the formation of autophagosome using a confocal laser scanning microscope. After matrix detachment, a greater number of LC3 puncta were induced in both control cells and the cells overexpressing miR-181a-5p. However, more LC3 puncta were observed in control cells compared with that in cells overexpressing miR-181a-5p (Fig. 2C). Together, these results affirm that autophagy induced in detachment condition was suppressed by miR-181a-5p.

**Figure 2:**
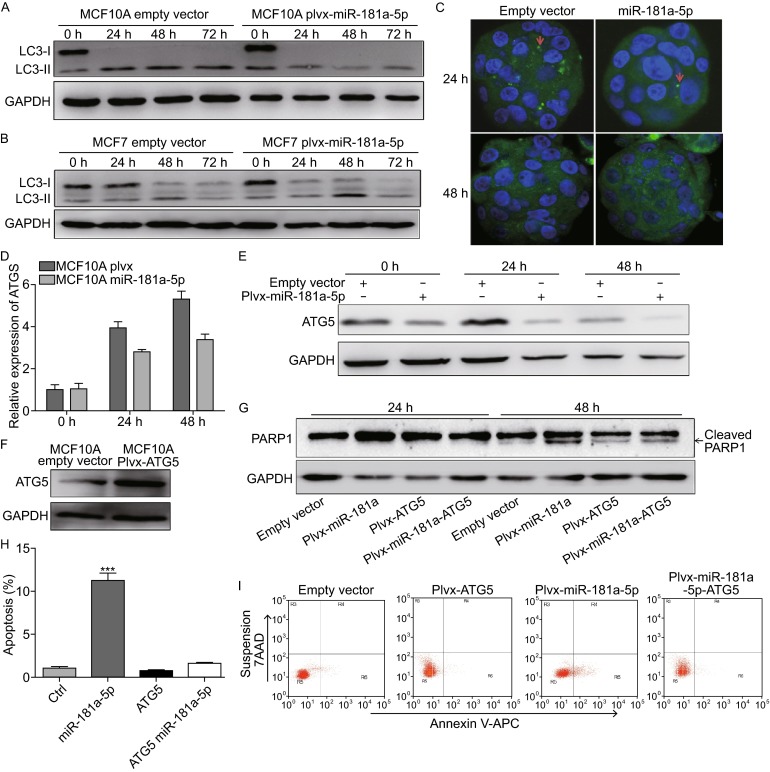
**MiR-181a-5p repressed anoikis resistance through blocked autophagy by targeting to** ***ATG5*** **in MCF10A during suspension culture**. (A and B) LC3 protein levels in MCF10A and MCF7 cells after detachment induction were detected by Western blot analysis. (C) LC3 Immunofluorescence. miR181a-5p blocked formation of suspension-induced LC3 dots in MCF10A cells. The autophagic activities of MCF10A cells with either empty vector or miR-181a-5p were assessed under detachment conditions for 24 h and 48 h. Arrows indicate the clusters of LC3 dots in cells. (D) miR181a-5p repressed the expression levels of ATG5 in MCF10A cells. (E) ATG5 protein levels were detected in MCF10A control cells with empty vector and miR-181a-5p-stable MCF10A cells. (F) ATG5 was overexpressed in MCF10A-plvx-ATG5 stable cell line. (G) Western blot analysis of the expression level of cleaved PARP1 in MCF10A cells transfected with empty vector, plvx-miR-181a-5p, plvx-ATG5, or the co-expression vector Plvx-miR-181a-ATG5, following suspension culture. (H and I) Apoptotic rates were detected using an Annexin V-APC kit at 48 h after detachment induction. For each cell line, 10,000 cells were analyzed by flow cytometry. The percentage of apoptosis was plotted by bar graph. The data correspond to the mean ± SD of three independent experiments (*P < 0.05)

miR-181a-5p promotes anoikis by repressing autophagy-related protein ATG5. Since miR-181a-5p can target ATG5 to inhibit autophagy induced by starvation (Tekirdag et al., 2013) and miR-181a-5p can repress autophagy in detachment culture condition, we explored whether miR-181a-5p targeted autophagy-related protein, ATG5, to promote anoikis in detachment-induced cells. As shown in Fig. 2D, in detachment condition, the expression of *ATG5* at mRNA level was downregulated in miR-181a-5p overexpressing MCF10A cells compared with that in control cells. Correspondingly, the expression of ATG5 at protein level also decreased in miR-181a-5p overexpressing cells (Fig. 2E). These results indicate that miR-181a-5p repressed the expression of *ATG5* in detachment cultures.

We further explored whether the repression of ATG5 by miR-181a-5p was resulted from the decreased anoikis in miR-181a-5p overexpressing MCF10A cells. The ATG5 overexpression cassette was constructed without the miR-181a-5p target site, leading to ATG5 expression that was insensitive to the regulation of miR-181a-5p. We then generated ATG5 overexpressing MCF10A cell line using this engineered ATG5 overexpression cassette. Western blot results exhibited that the expression of ATG5 at protein level was upregulated in MCF10A cells overexpressing *ATG5* compared with that in control cells (Fig. 2F). After 48 h of suspension culture, the expression level of cleaved PARP1 was much higher in cells stably-expressing miR-181a-5p compared with control cells as well as the cells overexpressing ATG5 (Fig. 2G). The expression level of cleaved PARP1 in cells co-expressing miR-181a-5p and ATG5 was similar to that in control cells (Fig. 2G). Furthermore, annexin V-APC/7-AAD staining indicated that the rate of detachment-induced apoptosis in MCF10A cells co-expressing ATG5 and miR-181a-5p was similar to that in both control cells and ATG5 overexpressing cells. In contrast, miR-181a-5p overexpressing MCF10A cells were more sensitive to detachment-induced apoptosis (Fig. 2H and 2I) (*P* < 0.01). The data indicate that miR-181a-5p-induced anoikis in MCF10A cells was rescued by the expression of ATG5, suggesting that miR-181a-5p promotes anoikis by repressing the expression of *ATG5*.

Previous studies have demonstrated that around twenty microRNAs play roles in the regulation of anoikis through different pathways in epithelial cells and cancer cells (Malagobadan and Nagoor, 2015). However, none of these miRNAs has been shown to target autophagy pathways in anoikis. In this study, we showed that miRNA miR-181a-5p promotes anoikis through inhibition of the expression of autophagy-related protein ATG5 in the mammary epithelial cell line MCF10A. Our data demonstrates that in detachment culture conditions, overexpression of miR-181a-5p promotes apoptosis and suppresses autophagy. We also show that restoration of *ATG5* expression inhibits the effects of miR-181a-5p on apoptosis and autophagy. It has been shown that several signaling pathways for anoikis resistance are activated when cells are detached from the ECM (Yang et al., 2013). Interestingly, inducing autophagy under ECM detachment conditions promotes cell survival, and autophagy has since been considered as an important energy supplying pathway in detached cells (Yang et al., 2013). Based on the knowledge that autophagy is involved in anoikis and our findings that autophagy is enhanced in detached MCF10A cells, we postulate that miR-181a-5p is able to inhibit autophagy through direct targeting of *ATG5* mRNA and that this pathway is largely responsible for the attenuation of anoikis resistance in normal mammary epithelial cell line, MCF10A. Furthermore, the fact that overexpression of miR-181a-5p promoted anoikis in MCF10A cells suggests that low levels of miR-181a-5p expression could indicate a risk for tumorigenesis, and that miR-181a-5p has the potential to be developed as an indicator in clinic diagnosis.

Investigating the regulators of anoikis resistance in cancer cells will undoubtedly contribute to advancements in cancer therapy. However, according to our findings, overexpression of miR-181a-5p in the breast cancer cell line, MCF7, did not repress anoikis resistance, unlike the effect observed in the normal mammary epithelial cell line, MCF10A. Autophagy was enhanced significantly in detached MCF10A cells, which supports the concept of autophagy as a survival mechanism in detached cells (Fung et al., 2008), however, it could not be promoted in MCF7 cells in which autophagy was not enhanced during detachment induction, implying that the anoikis was promoted by miR-181a-5p only in cell types with enhanced autophagy during anoikis. This suggests a possibility of differently regulated signaling pathways in anoikis resistance between the two cell lines, MCF10A and MCF7.

Our results indicate that miR-181a-5p acts as an antioncogenic gene, which has been identified as a dual-function miRNA involved in tumorigenesis in various cancer cell lines. Although previous studies have reported that miR-181a-5p is an antioncogene in non-small cell lung cancer (NSCLC) (Li et al., 2015; Niu et al., 2015), it has also been reported to act as an oncogene under drug-induced stress conditions in NSCLC (Li et al., 2015). As a result, the role of miR-181a-5p was condition-dependent in NSCLC. Similarly, miR-181a-5p seems to have different effects in distinct breast cell lines; apart from its function in MCF10A and MCF7, miR-181a-5p also promotes anoikis resistance by targeting Bim in breast tumors (Taylor et al., 2013). Furthermore, a recent study reported that miR-181a-5p was upregulated in triple-negative breast cancer (TNBC) cells that had undergone genotoxic treatments, and enhanced TNBC cell survival and metastasis (Niu et al., 2015). Together, it suggests that the therapeutic strategies employing miR-181a-5p in future should be developed based on the tumor types.

## Electronic supplementary material

Below is the link to the electronic supplementary material.

Supplementary material 1 (PDF 89 kb)
