# Supplementary material for: MiR-181a-5p promotes anoikis by suppressing autophagy during detachment induction in the mammary epithelial cell line MCF10A
Source: Protein Cell. 2016 Mar 14;7(4):305–9. doi: 10.1007/s13238-016-0255-8 (PMC4818842; doi:10.1007/s13238-016-0255-8)
Supplement: Supplementary file 1 — Supplementary material 1 (PDF 89 kb) [file 13238_2016_255_MOESM1_ESM.pdf]

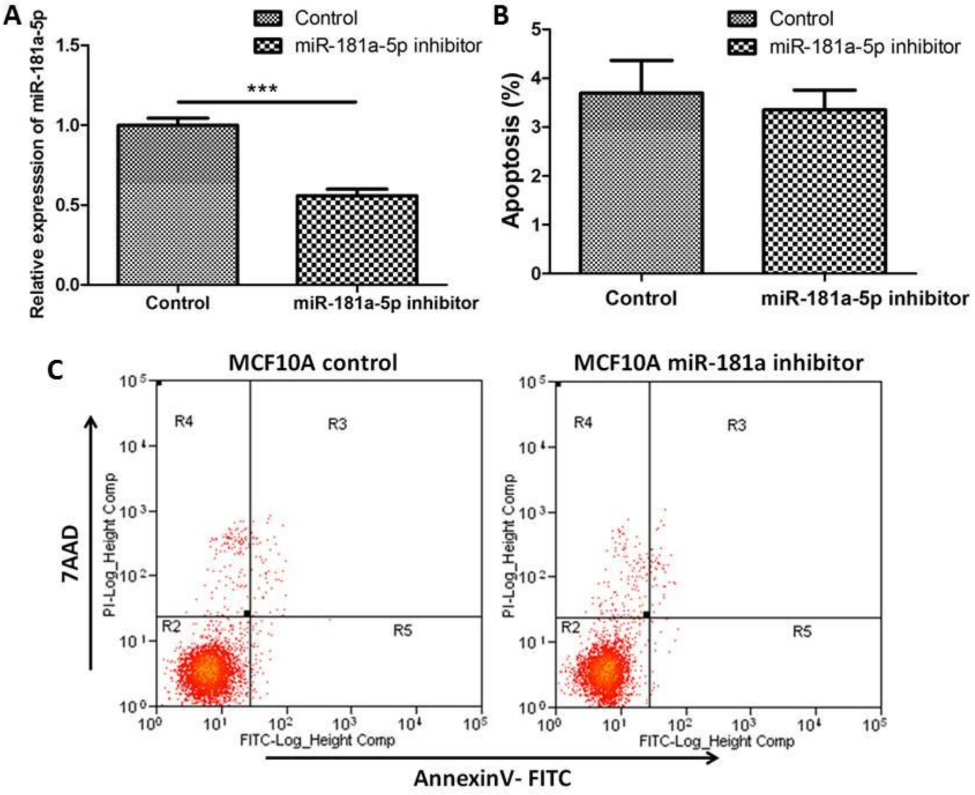

Figure S1. Knockdown the expression of miR-181a-5p had no effect on apoptosis during suspension. (A) miR-181a-5p inhibitor and control mimic were transfected into MCF10A. The expression of miR-181a-5p was knockdowned compared to control. (B and C) The apoptotic rate was detected with the Annexin V-APC kit following 48 h of suspension culture. For each cell line, 10000 cells were counted by flow cytometry.
